# Examining the potential causal relationships among smoking, blood DNA methylation, and type 2 diabetes development: A Mendelian randomization study

**DOI:** 10.18332/tid/216384

**Published:** 2026-07-17

**Authors:** Wenhua Li, Yixiao Li, Pan Dong, Jiaxin Tang, Siyang Liu, Ling Tu, Xizhen Xu

**Affiliations:** 1 Department of Geriatric Medicine, Tongji Hospital, Tongji Medical College, Huazhong University of Science and Technology, Wuhan, China; 2 Division of Cardiology and Department of Internal Medicine, Tongji Hospital, Tongji Medical College, Huazhong University of Science and Technology, Wuhan, China

**Keywords:** smoking, type 2 diabetes, DNA methylation, Mendelian randomization

## Abstract

**INTRODUCTION:**

Type 2 diabetes (T2D) is a major global health burden with rising prevalence and significant morbidity and mortality. Smoking has been recognized as an independent risk factor for T2D, but the exact underlying mechanisms are not yet fully understood.

**METHODS:**

We conducted a two-sample Mendelian randomization (MR) study to investigate the effects of smoking behaviors and smoking-related DNA methylation on type 2 diabetes (T2D) risk. This secondary analysis was based on publicly available summary statistics. Smoking traits were obtained from a large meta-GWAS of over 1.2 million individuals of European ancestry. DNA methylation data were derived from the CHARGE Consortium EWAS (n=15907) and mQTLs from the GoDMC (n=27750). T2D outcome data were obtained from a European GWAS including 74124 cases and 824006 controls. Replication was performed in FinnGen (17268 cases, 184778 controls) and BioBank Japan (40250 cases, 170615 controls). Stringently selected genetic instruments were applied, and causal effects were estimated primarily using two-sample MR with inverse-variance weighted analysis, complemented by MR-Egger and weighted median approaches, and further validated by colocalization analysis.

**RESULTS:**

In our genomic analysis, we presented convincing evidence supporting the association between genetically predicted smoking initiation and an increased susceptibility to T2D. In epigenetic analysis, we identified methylation at 21 smoking-related CpG sites associated with T2D risk. Subsequently, 14 of these CpG sites were validated using the FinnGen dataset, and 12 of them were validated using the BBJ dataset. Furthermore, we observed strong colocalization evidence for four CpG sites, including cg23756272 (*BCL2*), cg03864215 (*KCNJ11*), cg09861057 (*TDRD10*) and cg10672416 (*C12orf65*), and T2D risk.

**CONCLUSIONS:**

This research provides novel findings of the impact of smoking on T2D susceptibility, highlighting the role of epigenetic DNA methylation.

## INTRODUCTION

Type 2 diabetes (T2D) is a chronic metabolic disorder resulting from a complex interplay of genetic, epigenetic, and environmental factors. It is characterized by high prevalence and substantial morbidity and is a leading contributor to diminished quality of life and mortality. Smoking is not only significantly associated with T2D but also serves as an independent risk factor for the development of T2D^1^. Numerous observational studies have demonstrated that smokers have a higher risk of developing T2D compared to non-smokers^1,2^. Regular smokers exhibit a 15% to 30% elevated risk of developing T2D compared to individuals who have never smoked^3^. This heightened risk is dose-dependent, with the likelihood of developing T2D increasing with both smoking intensity and duration^1^. Nevertheless, due to the association of smoking with various lifestyle and socioeconomic factors, and the inherent challenges in controlling for all confounding variables in observational studies, the precise causal relationship between smoking and T2D remains incompletely understood. Importantly, the 2014 US Surgeon General’s Report concluded that smoking is a cause of type 2 diabetes, underscoring the urgent need to elucidate the underlying biological pathways^4^.

Mendelian randomization (MR) is an epidemiological approach that uses genetic variants as instrumental variables. MR analysis uses genetic variants to determine the causal effect of a risk factor on an outcome, and is considered advantageous over observational studies because it can mitigate confounding and reverse causation^5^.

With the advancement of epigenome-wide association studies (EWAS), smoking has been shown to be associated with widespread alterations in DNA methylation^6^. DNA methylation is a critical mechanism of epigenetic regulation and is thought to play a pivotal role in linking genetic and environmental factors in the pathogenesis and progression of T2D^7^. Walaszczyk et al.^8^ reported that methylation at multiple CpG sites and genes in blood samples was associated with T2D. Previous studies have also examined the link between smoking-related methylation and T2D. For example, Ligthart et al.^9^ reported that smoking was associated with differential methylation at several T2D susceptibility genes and that some of these methylation changes correlated with glycemic traits. However, this study was based on cross-sectional EWAS with relatively modest sample sizes and could not establish causality.

We postulated that DNA methylation may act as a responsive epigenetic mediator linking smoking exposure to T2D susceptibility. In this study, we employed a two-sample MR analysis to investigate the relationship between genetic predisposition to smoking behaviors and the risk of T2D, as well as to assess the influence of genetically predicted methylation at smoking-related CpG sites on T2D risk. Additionally, we further supported our findings through genetic colocalization analysis.

## METHODS

### Study design

**Figure 1:**
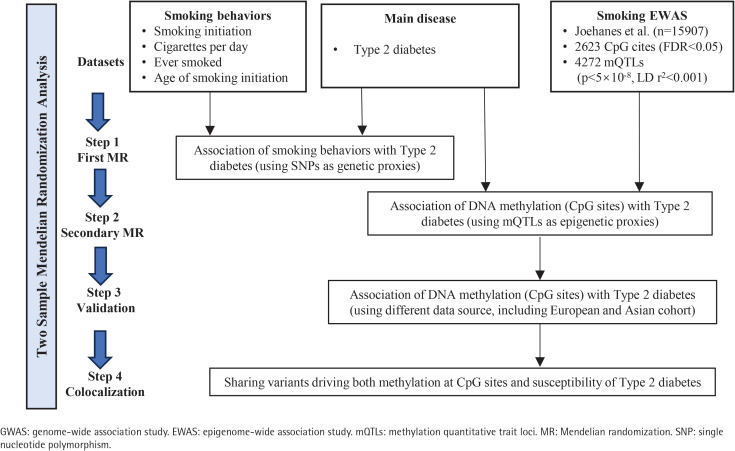
Study design schematic

This study employed a two-sample Mendelian randomization (MR) approach, utilizing summary statistics derived from genome-wide association studies (GWAS), as illustrated in Figure 1. The MR analysis was conducted under three principal assumptions: 1) Relevance – the instrumental variables (IVs) must be associated with the exposure, specifically smoking behaviors; 2) Exclusion restriction – the IVs should influence the outcome (T2D) only through the exposure; and 3) Independence – the IVs must be independent of any confounders affecting both the exposure and the outcome^5,10^. Initially, we conducted a two-sample MR analysis to evaluate the association between different smoking behaviors and the risk of type 2 diabetes. Subsequently, we investigated the relationship between smoking-related DNA methylation at CpG sites and the risk of type 2 diabetes. In the third step, significant CpG sites were validated using independent outcome data sources. Finally, a colocalization analysis was performed to ascertain whether the CpG sites and type 2 diabetes shared a common causal variant. This study followed the Strengthening the Reporting of Observational Studies in Epidemiology using Mendelian randomization (STROBE-MR) guidelines^11^. The STROBE-MR checklist is available in the Supplementary file. Details of the characteristics of the summary-level data obtained from genome-wide association studies (GWAS), which were conducted with ethical approval as authorized by the respective original studies, are given in Supplementary file Table S1.

### Exposure GWAS

We obtained summary statistics for smoking-related traits from the GWAS & Sequencing Consortium of Alcohol and Nicotine use (GSCAN), a large-scale meta-GWAS including over 1.2 million individuals of European ancestry^12^. Smoking initiation phenotypes were defined by two variables: age of initiation of regular smoking (age of smoking initiation, n=341427) and a binary variable indicating whether an individual had ever smoked regularly (smoking initiation, n=1232091). The heaviness of smoking was assessed through the number of cigarettes per day (cigarettes per day, n=337334), while the binary variable ever smoked (n=99996) compared current smokers to former smokers.

A comprehensive epigenome-wide association study (EWAS) meta-analysis conducted by the CHARGE Consortium identified 2623 CpG sites associated with smoking-related DNA methylation. This study analyzed 15907 blood-derived DNA samples across 16 cohorts, including 2433 current smokers, 6518 former smokers, and 6956 individuals who had never smoked^13^. To identify conditionally independent methylation quantitative trait loci (mQTLs), we utilized a catalog of SNPs provided by the Genetics of DNA Methylation Consortium (GoDMC). Methylation levels were quantified using the Illumina Infinium HumanMethylation450 BeadChip platform on bisulfite-converted genomic DNA across 36 cohorts, encompassing 27750 European samples sourced from whole blood^14^.

### Outcome GWAS

We obtained summary statistics for type 2 diabetes from the DIAMANTE consortium^15^, a European-ancestry GWAS comprising 74124 cases and 824006 controls across 32 studies. The effective sample size (Neff) of 231436 represents a 3.2-fold enhancement in Neff compared to the largest prior genome-wide study on type 2 diabetes risk in European populations^16^. To account for population structure and relatedness, association analyses were either adjusted for principal components (after the exclusion of related individuals) or implemented in a mixed model with random effects for kinship from a genetic relationship matrix.

Additionally, we included two additional datasets for replication. The first dataset was sourced from the FinnGen R10 cohort, comprising 17268 cases of type 2 diabetes and 184778 controls. FinnGen represents a substantial public-private collaboration aimed at the collection and analysis of genomic and health data from participants in Finnish biobanks. The genome-wide association study (GWAS) conducted within this dataset was adjusted for age, sex, ten principal components, and genotyping batch^17^. The second dataset utilized in this study was obtained from BioBank Japan (BBJ), which is recognized as one of the largest disease biobanks globally. Its primary objective is to facilitate research into personalized medical care tailored to individual patient characteristics. For this study, we included data comprising 40250 cases of type 2 diabetes and 170615 control subjects from the BBJ dataset^18^.

### Genetic instrument selection

In MR analyses, the genetic variants employed as instrumental variables for the exposure must be uncorrelated and exhibit a strong association (p<5×10^-8^) with the exposure of interest. From the SNPs identified in the GWAS of smoking behaviors and mQTLs, we further refined our selection to include only those variants with a minor allele frequency (MAF) >0.01. Additionally, we ensured that these SNPs were not in linkage disequilibrium (LD) (r^2^<0.001 and clumping window within 10000 base pairs, based on the European 1000 Genomes Project reference panel). This was done to meet the assumption of independence among the instrumental variables used in MR analyses. To mitigate the risk of weak instrument bias, we computed the F statistic for each instrumental variable, adhering to the widely accepted threshold^10,19^ of F>10.

### Statistical analysis

We performed a two-sample Mendelian randomization (MR) analysis to examine the relationship between smoking behaviors, smoking-related DNA methylation at CpG sites, and the risk of type 2 diabetes. The primary analytical approach employed was the inverse variance weighted (IVW) method under a multiplicative random-effects model. This random-effects IVW method assumes that all genetic variants are valid instruments and accounts for heterogeneity by aggregating SNP-specific Wald ratio estimates^20^. The results were presented as odds ratios (ORs) accompanied by 95% confidence intervals (CIs), indicating the risk of the disease per one standard deviation (SD) alteration in genetically predicted smoking behaviors or smoking-related DNA methylation at CpG sites. To address the issue of multiple testing, the Bonferroni correction was employed. Associations were deemed statistically significant if they had an adjusted p-value below the corrected threshold: p<0.0125 (0.05/4) for the four smoking behaviors, and p<2.42×10^-5^ (0.05/2063) for CpG sites, as determined by either the random-effects inverse-variance weighted (IVW) model or the Wald ratio model. All statistical tests were two-tailed.

To evaluate the third assumption of MR (the exclusion restriction criterion), we employed MR-Egger regression to identify potential violations arising from directional horizontal pleiotropy^21^. MR-Egger regression relies on the Instrument Strength Independent of Direct Effect (InSIDE) assumption, which requires that the association strength of genetic variants with the exposure is independent of any direct effects of the variants on the outcome. Furthermore, we utilized the weighted median method, which yields consistent estimates even when up to 50% of the instrumental variables are affected by pleiotropy^22^. Heterogeneity was examined using Cochran’s Q test in both the IVW and MR-Egger methods. The detailed results of heterogeneity tests are provided in Supplementary file Table S6.

For associations that remained significant after multiple-testing correction, we conducted a colocalization analysis utilizing the COLOC package in R. Colocalization analysis is instrumental in assessing whether a shared causal variant (PP.H4) is present between the disease genome-wide association study (GWAS) and the instruments for smoking-related DNA methylation at CpG sites. The analysis was executed using default priors (prior probability of association = 1×10^-4^, prior probability of shared causal variant = 1×10^-5^). A PP.H4 exceeding 85% was considered indicative of strong evidence for colocalization.

## RESULTS

### Genetically predicted smoking behaviors and the risk of T2D

Based on the criteria for selecting instrumental variables, a total of 129 single nucleotide polymorphisms (SNPs) were identified as instrumental variables for four distinct smoking behaviors. Specifically, 7 SNPs were selected for age of smoking initiation, 23 for cigarettes per day, 6 for ever smoked, and 93 for smoking initiation. The F-statistics for these instrumental variables ranged from 29.8 to 961, reflecting the strength of association between the SNPs and the corresponding smoking behaviors. Comprehensive details regarding the selected instrumental variables are provided in Supplementary file Table S2.

**Figure 2:**
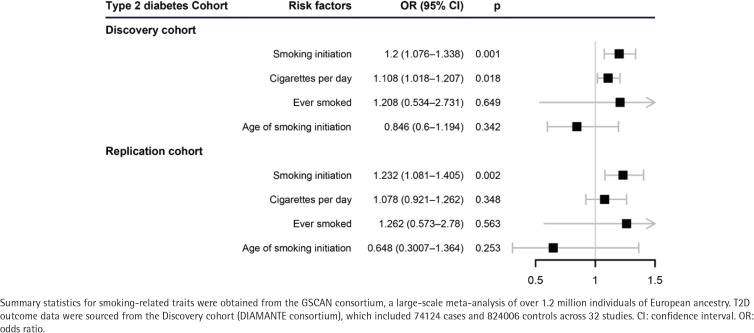
Mendelian randomization analysis of causal effects of smoking behaviors on type 2 diabetes

Our analyses using the inverse-variance Weighted (IVW) method indicated that genetically predicted smoking initiation was associated with an increased risk of T2D (OR=1.2; 95% CI: 1.076–1.338; p=0.001) (Figure 2), passing the Bonferroni-corrected threshold (p<0.0125). Consistent findings were observed using the weighted median method (OR=1.152; 95% CI: 1.045–1.27; p=0.004) (Supplementary file Table S3) and the simple median method (OR=1.17; 95% CI: 1.061–1.29; p=0.002) (Table Supplementary file S3). We also observed suggestive evidence of a positive association between genetically predicted cigarettes per day and the risk of T2D (OR=1.108; 95% CI: 1.018–1.207; p=0.018) (Figure 2), although this did not reach the Bonferroni-corrected significance threshold. However, we did not find a significant relationship between age of smoking initiation (OR=0.846; 95% CI: 0.6–1.194; p=0.342), ever smoked (OR=1.208; 95% CI: 0.534–2.731; p=0.649) and the risk of T2D (Figure 2).

There was also a significant positive association between genetically predicted smoking initiation and an increased risk of T2D in the FinnGen cohort (OR=1.232; 95% CI: 1.081–1.405; p=0.002) (Figure 2; and Supplementary file Table S3). In contrast, no significant associations were observed for genetically predicted age of smoking initiation (OR=0.648; 95% CI: 0.3007–1.364; p=0.253), cigarettes per day (OR=1.078; 95% CI: 0.921–1.262; p=0.348), and ever smoking status (OR=1.262; 95% CI: 0.573–2.78; p=0.563) with T2D risk (Figure 2; and Supplementary file Table S3).

### Genetically predicted smoking-related DNA methylation at CpG sites on T2D risk

To evaluate the causal impact of DNA methylation at smoking-related CpG sites on the risk of T2D, we referenced the identified methylation quantitative trait loci (mQTLs) from the Genetics of DNA Methylation Consortium (GoDMC) and performed two-sample MR and genetic colocalization analyses. Following the selection of instrumental variables, a total of 2063 smoking-related CpG sites were included for subsequent analysis in T2D. Consequently, we established a significance threshold of p<2.42×10^-5^ (0.05/2063) for T2D.

We identified that 21 smoking-related CpG sites were significantly associated with T2D, beyond a stringent Bonferroni threshold (Table 1; and Supplementary file Table S5). Of these, genetically predicted methylation at 14 smoking-related CpG sites was associated with an increased risk of T2D (Table 1; and Supplementary file Table S5), including cg23756272 (*BCL2*, OR=1.136; 95% CI: 1.082–1.193, p=3.26E-07), cg03864215 (*KCNJ11*, OR=1.133; 95% CI: 1.073–1.196, p=7.67E-06), cg03599224 (*LTA*, OR=1.116; 95% CI: 1.066–1.169, p=2.64E-06), cg17478749 (*SNORA38*; BAT*2*, OR=1.252; 95% CI: 1.132–1.384, p=1.17E-05), cg01300096 (*CUTA*, OR=1.631; 95% CI: 1.473–1.806, p=4.38E-21), cg09287933 (*CUTA*, OR=1.352; 95% CI: 1.215–1.504, p=2.99E-08), cg23657179 (*C10orf41*, OR=1.092; 95% CI: 1.059–1.127, p=3.31E-08), cg12864721 (*C10orf41*, OR=1.109; 95% CI: 1.069–1.150, p=4.20E-08), cg15182635 (*C10orf41*, OR=1.097; 95% CI 1.056–1.139, p=2.19E-06), cg20698113 (*PIM3*, OR=1.261; 95% CI: 1.151–1.382, p=6.72E-07), cg11801110 (*PIM3*, OR=1.196; 95% CI: 1.114–1.283, p=6.72E-07), cg10965178 (*TIE1*, OR=1.400; 95% CI: 1.272–1.541, p=5.37E-12), cg10672416 (*C12orf65*, OR=1.105; 95% CI: 1.065–1.147, p=1.13E-07), and cg09861057 (*TDRD10*, OR=1.375; 95% CI: 1.206–1.568, p=1.98E-06) (Table 1; and Supplementary file Table S5).

**Table 1:** Two-sample Mendelian randomization of smoking-related DNA methylation at CpG sites and the risk of type 2 diabetes

*Exposure*	*Method*	*NSNP*	*β*	*SE*	*p*	*Adjusted p*
cg01300096	Inverse variance weighted	2	0.489	0.052	4.38E-21	9.04E-18
cg07123182	Wald ratio	1	-0.183	0.022	2.53E-17	5.21E-14
cg01744331	Wald ratio	1	-0.180	0.021	2.53E-17	5.21E-14
cg16556677	Wald ratio	1	-0.185	0.022	2.53E-17	5.21E-14
cg10965178	Inverse variance weighted	2	0.337	0.049	5.37E-12	1.11E-08
cg24142464	Wald ratio	1	-0.343	0.052	5.80E-11	1.20E-07
cg26963277	Inverse variance weighted	2	-0.184	0.030	1.08E-09	2.23E-06
cg09287933	Wald ratio	1	0.301	0.054	2.99E-08	6.16E-05
cg23657179	Inverse variance weighted	3	0.088	0.016	3.31E-08	6.83E-05
cg12864721	Inverse variance weighted	3	0.103	0.019	4.20E-08	8.66E-05
cg10672416	Wald ratio	1	0.100	0.019	1.13E-07	2.33E-04
cg15948030	Inverse variance weighted	2	-0.438	0.083	1.56E-07	3.22E-04
cg23756272	Wald ratio	1	0.128	0.025	3.26E-07	6.72E-04
cg20698113	Wald ratio	1	0.232	0.047	6.72E-07	1.39E-03
cg11801110	Wald ratio	1	0.179	0.036	6.72E-07	1.39E-03
cg09861057	Wald ratio	1	0.318	0.067	1.98E-06	4.09E-03
cg15182635	Inverse variance weighted	3	0.092	0.019	2.19E-06	4.52E-03
cg03599224	Inverse variance weighted	2	0.110	0.023	2.64E-06	5.44E-03
cg03864215	Inverse variance weighted	2	0.125	0.028	7.67E-06	1.58E-02
cg20069688	Wald ratio	1	-0.111	0.025	1.15E-05	2.38E-02
cg17478749	Inverse variance weighted	3	0.224	0.051	1.17E-05	2.40E-02

DNA methylation associations were obtained from the CHARGE consortium EWAS (n=15907). Type 2 diabetes (T2D) outcome data were sourced from the DIAMANTE consortium, which included 74124 cases and 824006 controls across 32 studies.

In contrast, methylation at the remaining seven CpG sites was significantly related to a decreased risk of T2D (Table 1; and Supplementary file Table S5), including cg07123182 (*KCNQ1OT1*; *KCNQ1*, OR=0.833; 95% CI: 0.798–0.869, p=2.53E-17), cg01744331 (*KCNQ1OT1*; *KCNQ1*, OR=0.835; 95% CI: 0.801–0.871, p=2.53E-17), cg16556677 (*KCNQ1OT1*; *KCNQ1*, OR=0.831; 95% CI: 0.796–0.867, p=2.53E-17), cg26963277 (*KCNQ1OT1*; *KCNQ1*, OR=0.832; 95% CI: 0.785–0.883, p=1.08E-09), cg24142464 (*PABPC4*, OR=0.709; 95% CI: 0.640-0.786, p=5.80E-11), cg15948030 (*VARS*, OR=0.646; 95% CI: 0.548–0.760, p=1.56E-07) and cg20069688 (*STK19*; *DOM3Z*, OR=0.895; 95% CI: 0.851–0.940, p=1.15E-05) (Table 1; and Supplementary file Table S5).

### Replication results

**Figure 3:**
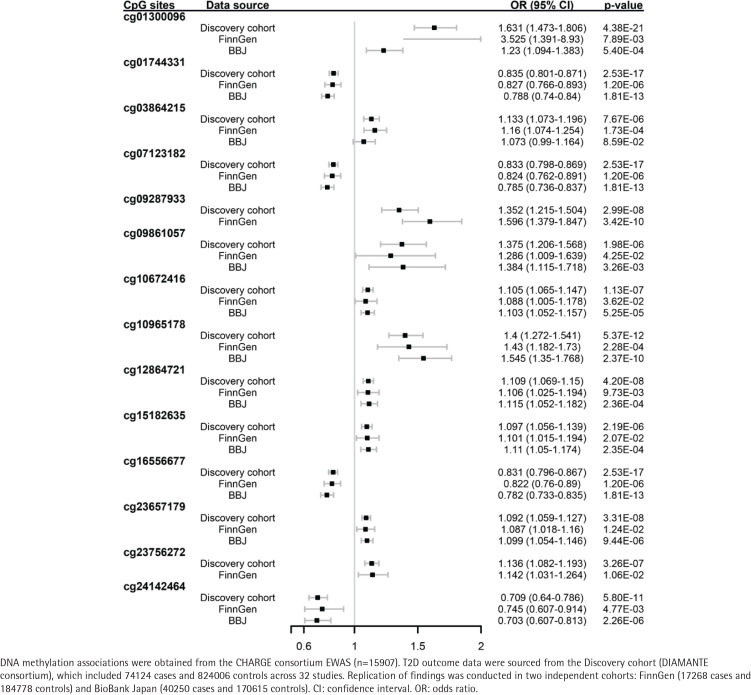
Mendelian Randomization analysis of smoking-related DNA methylation at CpG sites and T2D risk: replication in FinnGen and BioBank Japan

To further validate our findings, we included independent datasets for further analysis. In the FinnGen cohort, a total of 14 CpG sites were replicated (Figure 3). Of these, genetically predicted methylation at 10 smoking-related CpG sites was associated with an increased risk of T2D, while the remaining four CpG sites were significantly associated with a reduced risk of T2D (Figure 3).

To assess these associations in an Asian population, we utilized GWAS data from BBJ and conducted MR analyses. We replicated associations for 12 CpG sites with T2D risk (Figure 3). Specifically, genetically predicted methylation at 8 CpG sites was significantly associated with an increased risk of T2D, while the remaining 4 CpG sites were associated with a decreased risk of T2D (Figure 3).

Additionally, we identified robust evidence of colocalization for four CpG sites, including cg23756272 (*BCL2*, PP.H4=0.998), cg03864215 (*KCNJ11*, PP.H4=0.977), cg09861057 (*TDRD10*, PP.H4=0.931), and cg10672416 (*C12orf65*, PP.H4=0.89), which exhibited significant associations with T2D risk (Table 2).

**Table 2:** The results of colocalization analysis

*Exposure*	*PP.H0*	*PP.H1*	*PP.H2*	*PP.H3*	*PP.H4*
cg23756272	1.58E-174	6.74E-04	5.66E-174	1.42E-03	9.98E-01
cg03864215	1.06E-116	2.30E-02	5.37E-118	1.95E-04	9.77E-01
cg09861057	1.14E-27	5.57E-03	1.32E-26	6.36E-02	9.31E-01
cg10672416	0.00E+00	2.24E-04	0.00E+00	1.10E-01	8.90E-01

DNA methylation associations were obtained from the CHARGE consortium EWAS (n=15907) and from methylation quantitative trait loci (mQTLs) identified by the GoDMC consortium (n=27750 across 36 European cohorts). T2D outcome data were sourced from the DIAMANTE consortium, which included 74124 cases and 824006 controls across 32 studies.

## DISCUSSION

In this study, we examined the relationship between smoking behaviors and T2D risk at the genomic and epigenomic levels using MR analyses, and validated these findings through genetic colocalization.

Our genomic analysis provided convincing evidence supporting the causal impact of smoking initiation on T2D. Smoking has been established as an independent risk factor for T2D^1^. Our genomic MR analysis indicated a positive association between genetically predicted smoking initiation and increased T2D risk, with suggestive evidence for cigarettes per day, but no significant associations for ever smoked or age of smoking initiation. Moreover, this association was replicated in the FinnGen cohort.

When exploring the effect of genetically predicted methylation at smoking-related CpG sites on T2D risk, we found that 21 CpG sites were significantly associated with T2D risk, suggesting potential epigenetic regulation. Of these, 14 were associated with increased risk, whereas 7 were associated with decreased risk. Among these sites, 14 were replicated in FinnGen and 12 in BBJ. In addition, we performed genetic colocalization analysis and found that four CpG sites, including cg23756272 (*BCL2*), cg03864215 (*KCNJ11*), cg09861057 (*TDRD10*), and cg10672416 (*C12orf65*), had strong colocalization evidence.

Apoptosis plays a critical role in the pathophysiology of T2D. The B-cell lymphoma-2 (*BCL2*) gene, a member of the *BCL-2* family, has well-established anti-apoptotic functions^23^. A comprehensive meta-analysis of 39 studies across multiple ethnic groups identified a significant association between the *BCL2* variant rs12454712T and T2D^24^. Our study identified that methylation at cg23756272 (*BCL2*) was associated with an increased risk of T2D, and this finding was replicated in the FinnGen cohort. Genetic colocalization analysis also showed strong colocalization evidence for cg23756272 methylation and T2D susceptibility. Collectively, our study provides new insights into the role of *BCL-2* in the development of T2D from the perspective of DNA methylation.

The *KCNJ11* gene encodes the inward rectifier potassium ion channel, Kir6.2, which constitutes a classical ATP-sensitive potassium (KATP) channel through its interaction with the sulfonylurea receptor^25^. *KCNJ11* plays a crucial role in the regulation of insulin secretion in pancreatic β-cells. Different mutation sites of the *KCNJ11* gene led to a series of continuous and varying degrees of glucose metabolism abnormalities including T2D^26^. The *KCNJ11* E23K gene polymorphism was significantly associated with the onset of T2D and was recognized as one of the susceptibility genes for T2D^26^. Our study identified that methylation at cg03864215 (*KCNJ11*) was associated with an increased risk of T2D, and this finding was replicated in the FinnGen cohort. Genetic colocalization analysis further provided strong evidence linking cg03864215 methylation to T2D susceptibility. Collectively, these findings indicate that *KCNJ11* contributes to the pathogenesis of T2D and underscore the need for further investigations into its specific role in T2D development.

The *LTA* gene encodes lymphotoxin-alpha (LT-α), also referred to as tumor necrosis factor-beta (TNF-β), a proinflammatory cytokine predominantly produced by lymphocytes^27^. Polymorphisms in *LTA* have been significantly associated with T2D susceptibility at the population level. In Japanese men, the 252A→G and 804C→A variants of *LTA* are associated with T2D risk^28^. In White populations, *LTA* variants have likewise been associated with increased T2D risk and features of the metabolic syndrome^29^. Building on this evidence, we found that DNA methylation at cg03599224 within *LTA* is associated with a higher risk of T2D, suggesting that *LTA* may contribute to T2D pathogenesis through both genetic and epigenetic mechanisms.

Our study showed that methylation at cg17478749 (*SNORA38*; *BAT2*) was associated with an increased risk of T2D. *BAT2*, also referred to as *PPRC2A*, is located within the class III region of the human major histocompatibility complex. Kong et al.^30^ reported that the BAT2 rs2260000 variant influences T2D risk in the Chinese Han population independently of obesity. Research on the *SNORA38* gene and its association with T2D risk is limited. Our findings indicate that epigenetic dysregulation of *SNORA38* may play a mediating role in the pathogenic impact of smoking on T2D development. Further experimental investigations are warranted to substantiate these findings.

The potassium voltage-gated channel KQT-like subfamily Q member 1 (*KCNQ1*) gene encodes the pore-forming α subunit of the IKs K^+^ channel^31^ and has been recognized as a susceptibility gene for T2D^31,32^. SNPs in *KCNQ1* have been linked to an increased risk of T2D in East Asian and European populations. The long non-coding RNA *KCNQ1* overlapping transcript 1 (*KCNQ1OT1*), located at the *KCNQ1* locus^33^, has also been implicated in T2D, with a meta-analysis demonstrating a significant association between the rs231362 polymorphism and T2D risk^34^. In our study, methylation at four CpG sites in *KCNQ1OT1* and *KCNQ1*, including cg07123182, cg01744331, cg16556677, and cg26963277, was associated with a reduced risk of T2D; notably, cg07123182 was replicated in both the FinnGen and BBJ databases, while cg01744331 and cg16556677 were replicated in FinnGen. Collectively, these findings suggest that *KCNQ1OT1* and *KCNQ1* contribute to T2D pathogenesis through both genetic susceptibility and epigenetic regulation.

Previous studies have suggested a potential role of *PABPC4* in T2D. In human studies, *PABPC4* has been associated with T2D risk through modulation of cg15123755 methylation and effects on high-density lipoprotein levels^35^. In our study, methylation at cg24142464 (*PABPC4*) was significantly associated with reduced T2D risk. Future investigations into the interactions between smoking, epigenetic modifications, and functional alterations of *PABPC4*, and their role in T2D development and progression, will be of considerable importance.

Additionally, our study found that methylation at cg01300096 (*CUTA*), cg09287933 (*CUTA*), cg10965178 (*TIE1*), cg23657179 (*C10orf41*), cg15182635 (*C10orf41*), cg12864721 (*C10orf41*), cg10672416 (*C12orf65*), cg20698113 (*PIM3*), cg11801110 (*PIM3*), and cg09861057 (*TDRD10*) was associated with an increased risk of T2D, whereas methylation at cg15948030 (*VARS*) and cg20069688 (*STK19*; *DOM3Z*) was associated with a decreased risk. Although limited research has examined the associations of these genes with T2D, our study provides robust evidence, based on mQTLs, supporting their linkage to T2D risk. Future functional studies will be crucial to validate our findings and elucidate the roles of these genes in T2D pathogenesis.

### Strengths and limitations

There are several strengths to our study. Firstly, we employed a two-sample MR analysis to evaluate the causal relationship between smoking behaviors, smoking-related blood DNA methylation, and the risk of T2D. This approach enhances the reliability of causal inference by mitigating residual confounding and reverse causality^5^. Additionally, we investigated the association between genetically predicted methylation at smoking-related CpG sites and T2D risk across diverse independent datasets, thereby augmenting the generalizability of the population and the validity of our findings.

There are also some limitations to our study. Firstly, the DNA methylation data for smoking-related CpG sites were derived from cross-sectional EWAS, which precludes the assessment of the temporal progression, kinetics, and dynamics of smoking’s impact on DNA methylation^36^. Consequently, longitudinal studies are needed to clarify causality over time. Second, our analysis was based on blood-derived DNA methylation patterns, which may differ substantially across tissues. Third, as our analyses relied on publicly available GWAS summary statistics, potential sample overlap at the individual level cannot be fully excluded. However, replication in FinnGen and BioBank Japan, along with colocalization analysis, reduces the likelihood of substantial bias. Fourth, although population stratification was controlled for in the original GWAS analyses through principal component analysis and mixed models, residual confounding due to unaccounted population structure may still remain, especially when combining datasets from different populations. Fifth, the lack of East Asian-specific mQTL data may lead to population stratification or weak instrument bias in cross-population replication. While the FinnGen dataset was used for primary validation, which is ancestry-matched to the mQTLs, the BBJ replication analysis was exploratory. The absence of ancestry-matched mQTL data in East Asians limits the interpretation of cross-population results. Future studies incorporating ancestry-matched QTL datasets are essential for improving the reliability and generalizability of findings. Sixth, Mendelian randomization itself has inherent limitations, including the inability to completely rule out unobserved horizontal pleiotropy; therefore, causal inferences should be interpreted as suggestive rather than definitive. Seventh, our primary analyses were based on European populations and replicated in Finnish and Japanese cohorts; however, differences in genetic architecture, lifestyle, and environmental exposures between European and Asian populations may affect the transferability of findings. Finally, the lack of replication in non-European populations (e.g. African or Latin American cohorts) limits the generalizability of our conclusions.

## CONCLUSIONS

This study provides further evidence of the potential pathogenic role of smoking in T2D development. Our findings support a significant association between smoking and T2D risk. Furthermore, the evidence indicates that alterations in DNA methylation at specific CpG sites and associated genes may play a crucial mediating role in this relationship.

## Supplementary Material



## Data Availability

The data supporting this research are available from the authors on reasonable request.
