# Supplementary material for: Examining the potential causal relationships among smoking, blood DNA methylation, and type 2 diabetes development: A Mendelian randomization study
Source: Tob Induc Dis. 2026 Jul 17;24:10.18332/tid/216384. doi: 10.18332/tid/216384 (PMC13386699; doi:10.18332/tid/216384)
Supplement: Supplementary file 1 [file TID-24-113-s1.pdf]

TID
Tobacco Induced Diseases

Supplementary file

© 2026 Li W. et al.

DOI:
10.18332/tid/216384

The content has been provided by the author(s) and has not been reviewed, verified, or endorsed by European Publishing. It may not have undergone peer review. The views, opinions, and recommendations expressed are solely those of the author(s) and do not necessarily reflect the position of European Publishing. European Publishing accepts no responsibility or liability for any consequences arising from the use of, or reliance on, this content.

Table S1: Datasets used in the study

Trait	PMID	Sample size	Population
Age of smoking initiation	30643251	341427	European
Cigarettes per day	30643251	337334	European
Smoking initiation	30643251	1232091	European
Ever smoked		99996	European
mQTLs	34493871	27750	European
Type 2 diabetes-discovery cohort	30297969	898130	European
Type 2 diabetes-FinnGen		202046	European
Type 2 diabetes-BBJ		210865	East Asian

Table S2: Genetic instruments of smoking behaviors used in this Mendelian randomization study

exposure	SNP	effect_allele.exposure	other_allele.exposure	beta.exposure	se.exposure	pval.exposure	F
Age Of Smoking Initiation	rs10200107	A	G	-0,01956	0,002777	1,84E-12	49,6
Age Of Smoking Initiation	rs3768886	C	G	0,01714	0,002966	7,37E-09	33,4
Age Of Smoking Initiation	rs11915747	G	C	0,02099	0,002891	3,83E-13	52,7
Age Of Smoking Initiation	rs624833	G	T	0,017295	0,003006	8,61E-09	33,1
Age Of Smoking Initiation	rs11780471	A	G	0,03785	0,005806	7,00E-11	42,5
Age Of Smoking Initiation	rs140485736	A	G	0,065453	0,011535	1,41E-08	32,2
Age Of Smoking Initiation	rs319748	A	G	-0,01703	0,003074	3,08E-08	30,7
smoking initiation	rs3001723	A	G	0,033512	0,003898	8,12E-18	73,9
smoking initiation	rs7555507	T	C	-0,02414	0,003556	1,14E-11	46,1
smoking initiation	rs6669839	T	C	0,026004	0,004396	3,36E-09	35
smoking initiation	rs12042107	C	T	-0,02228	0,003568	4,22E-10	39
smoking initiation	rs2186122	T	A	0,026057	0,003586	3,61E-13	52,8
smoking initiation	rs301805	G	T	0,021468	0,003613	2,80E-09	35,3
smoking initiation	rs12025237	C	A	-0,033	0,005339	6,52E-10	38,2
smoking initiation	rs2050586	C	G	-0,02055	0,003708	3,00E-08	30,7
smoking initiation	rs2046850	T	C	-0,02481	0,004478	3,03E-08	30,7
smoking initiation	rs6728726	C	T	0,035449	0,004733	6,73E-14	56,1
smoking initiation	rs78411160	C	A	0,020536	0,003659	2,03E-08	31,5
smoking initiation	rs6433897	C	T	0,022448	0,004058	3,16E-08	30,6
smoking initiation	rs266047	A	G	-0,03051	0,003739	3,36E-16	66,6
smoking initiation	rs4674993	G	A	-0,02521	0,004436	1,32E-08	32,3
smoking initiation	rs578584	T	A	0,02868	0,003596	1,50E-15	63,6
smoking initiation	rs35702515	T	G	0,025244	0,004231	2,43E-09	35,6
smoking initiation	rs13030994	A	G	0,036093	0,003556	3,56E-24	103
smoking initiation	rs12474587	T	G	0,027633	0,003582	1,25E-14	59,5
smoking initiation	rs2107300	G	C	-0,0272	0,004925	3,27E-08	30,5
smoking initiation	rs7585579	G	C	0,0224	0,003728	1,88E-09	36,1
smoking initiation	rs1445649	C	T	0,023993	0,003565	1,68E-11	45,3
smoking initiation	rs6788098	T	A	-0,03135	0,003689	1,91E-17	72,2
smoking initiation	rs12632110	G	A	-0,02338	0,003753	4,78E-10	38,8
smoking initiation	rs11712680	C	A	-0,02705	0,004578	3,51E-09	34,9
smoking initiation	rs1154693	G	A	0,032622	0,004912	3,12E-11	44,1
smoking initiation	rs66680800	T	G	-0,02027	0,003653	2,83E-08	30,8
smoking initiation	rs1869243	C	T	0,019741	0,003563	2,97E-08	30,7
smoking initiation	rs9835772	T	A	0,024047	0,004142	6,32E-09	33,7
smoking initiation	rs962625	G	A	0,023718	0,004038	4,37E-09	34,5
smoking initiation	rs993700	C	T	-0,02593	0,004292	1,53E-09	36,5
smoking initiation	rs13145728	C	G	-0,02325	0,003663	2,14E-10	40,3
smoking initiation	rs10001365	A	G	-0,02499	0,003642	6,65E-12	47,1
smoking initiation	rs1160685	G	C	0,020772	0,003589	7,20E-09	33,5
smoking initiation	rs6893752	G	A	-0,0241	0,004074	3,25E-09	35
smoking initiation	rs12186738	T	G	-0,03326	0,005021	3,42E-11	43,9
smoking initiation	rs1385108	T	C	0,024662	0,004157	3,00E-09	35,2
smoking initiation	rs4044321	G	A	-0,02784	0,003711	6,08E-14	56,3
smoking initiation	rs4352629	T	C	-0,02753	0,003569	1,22E-14	59,5
smoking initiation	rs72789632	T	C	-0,03289	0,005286	5,02E-10	38,7
smoking initiation	rs9401770	A	G	0,027731	0,003986	3,47E-12	48,4
smoking initiation	rs222449	T	A	-0,02532	0,004428	1,08E-08	32,7
smoking initiation	rs3800227	G	A	0,022812	0,004058	1,93E-08	31,6
smoking initiation	rs10498846	T	C	0,02061	0,003556	6,62E-09	33,6
smoking initiation	rs240963	C	T	-0,04104	0,004837	2,16E-17	72
smoking initiation	rs12333760	C	T	-0,02905	0,004801	1,44E-09	36,6
smoking initiation	rs10233018	G	A	0,027069	0,003557	2,75E-14	57,9
smoking initiation	rs10279261	A	G	-0,02142	0,003663	5,00E-09	34,2
smoking initiation	rs10260968	A	G	-0,02032	0,003609	1,75E-08	31,7
smoking initiation	rs12112638	G	A	-0,02453	0,004043	1,34E-09	36,8
smoking initiation	rs4236259	G	T	-0,02477	0,003557	3,35E-12	48,5
smoking initiation	rs2140114	T	C	-0,02326	0,003734	4,70E-10	38,8
smoking initiation	rs3801289	C	A	-0,02206	0,00374	3,74E-09	34,8
smoking initiation	rs1565735	A	T	-0,03762	0,004461	3,42E-17	71,1
smoking initiation	rs1899896	T	C	0,026448	0,003887	1,04E-11	46,3
smoking initiation	rs13261666	T	G	-0,02689	0,003556	3,90E-14	57,2
smoking initiation	rs12545053	G	A	0,020281	0,003637	2,43E-08	31,1
smoking initiation	rs2631024	G	A	-0,02296	0,004028	1,18E-08	32,5
smoking initiation	rs4543592	C	T	0,021931	0,003562	7,46E-10	37,9
smoking initiation	rs2378662	A	G	0,020948	0,003566	4,16E-09	34,5
smoking initiation	rs10114490	A	G	-0,02551	0,004532	1,81E-08	31,7
smoking initiation	rs10905461	C	T	-0,02396	0,004145	7,35E-09	33,4
smoking initiation	rs7921378	C	G	-0,02546	0,003558	8,26E-13	51,2
smoking initiation	rs12356821	C	G	0,03937	0,005049	6,27E-15	60,8
smoking initiation	rs10159545	G	C	0,02625	0,003727	1,84E-12	49,6
smoking initiation	rs9423279	G	C	-0,02051	0,003708	3,21E-08	30,6
smoking initiation	rs7938812	G	T	0,043791	0,003637	2,71E-33	145
smoking initiation	rs6265	T	C	-0,03179	0,004578	3,77E-12	48,2
smoking initiation	rs7929518	G	A	0,024238	0,004285	1,56E-08	32
smoking initiation	rs4523689	G	A	-0,02061	0,003643	1,55E-08	32
smoking initiation	rs11057005	G	A	-0,02093	0,003579	4,85E-09	34,2
smoking initiation	rs4759228	C	G	-0,02169	0,003934	3,58E-08	30,4
smoking initiation	rs7969559	G	A	-0,02438	0,003959	7,31E-10	37,9
smoking initiation	rs1971318	T	C	0,028507	0,004925	7,06E-09	33,5
smoking initiation	rs7322872	T	C	-0,02557	0,004335	3,58E-09	34,8
smoking initiation	rs3904512	A	G	-0,02116	0,003577	3,23E-09	35
smoking initiation	rs9540729	T	A	-0,01955	0,003558	3,82E-08	30,2
smoking initiation	rs76214862	C	A	-0,02499	0,004547	3,99E-08	30,2
smoking initiation	rs12441907	A	C	-0,02921	0,004523	1,06E-10	41,7
smoking initiation	rs1435741	A	G	0,029415	0,003591	2,64E-16	67,1
smoking initiation	rs4785836	C	T	-0,02047	0,003659	2,26E-08	31,3
smoking initiation	rs7197072	T	C	-0,02477	0,004169	2,77E-09	35,3
smoking initiation	rs1050847	T	C	-0,02162	0,003589	1,67E-09	36,3
smoking initiation	rs4781977	C	T	-0,02387	0,004365	4,54E-08	29,9
smoking initiation	rs11078713	G	A	-0,02017	0,003606	2,23E-08	31,3
smoking initiation	rs7224742	T	C	-0,02071	0,003655	1,43E-08	32,1
smoking initiation	rs11658881	G	A	0,020136	0,003611	2,43E-08	31,1
smoking initiation	rs6508144	G	C	-0,02069	0,003586	7,97E-09	33,3
smoking initiation	rs11872397	A	G	-0,02477	0,004095	1,43E-09	36,6
smoking initiation	rs72896886	C	G	-0,02689	0,004837	2,75E-08	30,9
smoking initiation	rs76608582	A	C	-0,04956	0,00826	1,94E-09	36
smoking initiation	rs1555445	T	A	0,022555	0,003823	3,65E-09	34,8
smoking initiation	rs117143374	C	T	0,02929	0,005269	2,76E-08	30,9
smoking initiation	rs134529	C	T	-0,01998	0,003661	4,85E-08	29,8
Cigarettes smoked per day	rs2072659	G	C	-0,06525	0,009247	1,71E-12	49,8
Cigarettes smoked per day	rs2084533	T	C	0,033641	0,005901	1,22E-08	32,5
Cigarettes smoked per day	rs7431710	A	G	-0,03496	0,00581	1,82E-09	36,2
Cigarettes smoked per day	rs787362	A	T	0,030477	0,005574	4,50E-08	29,9
Cigarettes smoked per day	rs11725618	C	T	0,036064	0,006158	4,67E-09	34,3
Cigarettes smoked per day	rs806798	C	T	-0,03085	0,005532	2,48E-08	31,1
Cigarettes smoked per day	rs215600	A	G	-0,04925	0,005753	1,10E-17	73,3
Cigarettes smoked per day	rs73229090	A	C	0,05549	0,008763	2,44E-10	40,1
Cigarettes smoked per day	rs790564	C	A	-0,04089	0,006193	3,97E-11	43,6
Cigarettes smoked per day	rs58379124	C	T	0,066939	0,006502	9,00E-25	106
Cigarettes smoked per day	rs3025383	C	T	-0,05784	0,007045	2,22E-16	67,4
Cigarettes smoked per day	rs7951365	C	T	0,038951	0,005968	6,63E-11	42,6
Cigarettes smoked per day	rs75494138	T	C	0,059876	0,010568	1,45E-08	32,1
Cigarettes smoked per day	rs7928017	A	C	-0,03293	0,005558	3,14E-09	35,1
Cigarettes smoked per day	rs632811	G	A	-0,03671	0,00641	1,03E-08	32,8
Cigarettes smoked per day	rs8034191	C	T	0,182567	0,005889	1,00E-200	961
Cigarettes smoked per day	rs4785587	A	G	-0,03362	0,005535	1,27E-09	36,9
Cigarettes smoked per day	rs1579233	G	A	-0,03182	0,005565	1,07E-08	32,7
Cigarettes smoked per day	rs895330	G	C	-0,039	0,007017	2,68E-08	30,9
Cigarettes smoked per day	rs56113850	C	T	0,107205	0,005604	1,10E-81	366
Cigarettes smoked per day	rs34406232	A	C	-0,14699	0,016697	1,33E-18	77,5
Cigarettes smoked per day	rs2273500	C	T	0,068094	0,007796	2,47E-18	76,3
Cigarettes smoked per day	rs2424888	A	G	0,033485	0,005636	2,76E-09	35,3
Ever smoked	rs12731986	T	C	-0,0121	0,0022	2,17E-08	30,3
Ever smoked	rs9287372	G	A	-0,0148	0,0021	4,05E-12	49,7
Ever smoked	rs846184	T	C	0,0179	0,0032	1,54E-08	31,3
Ever smoked	rs7829715	C	T	-0,0125	0,0021	4,12E-09	35,4
Ever smoked	rs2011487	A	C	-0,0142	0,0021	3,96E-11	45,7
Ever smoked	rs12874797	T	C	-0,0194	0,0034	1,06E-08	32,6

Table S3: Two-sample Mendelian randomization of smoking behaviors with type 2 diabetes

Cohort	outcome	exposure	method	nsnp	b	se	pval	or	or_lci95	or_uci95	Egger_intercept	Egger_se	Egger_pval	MR Egger-Q_pval	Inverse variance weighted-Q_pval
Discovery cohort	Type 2 diabetes	Age Of Smoking Initiation	MR Egger	6	0,325	0,58	0,605	1,384	0,444	4,316	-0,0104	0,0117	0,4242	0,928	0,8932
Discovery cohort	Type 2 diabetes	Age Of Smoking Initiation	Weighted median	6	-0,101	0,215	0,638	0,904	0,592	1,379					
Discovery cohort	Type 2 diabetes	Age Of Smoking Initiation	Inverse variance weighted	6	-0,167	0,176	0,342	0,846	0,6	1,194					
Discovery cohort	Type 2 diabetes	Age Of Smoking Initiation	Simple median	6	-0,132	0,213	0,535	0,876	0,577	1,331					
Discovery cohort	Type 2 diabetes	Cigarettes smoked per day	MR Egger	22	-0,08	0,066	0,234	0,923	0,812	1,049	0,0135	0,0041	0,0033	0,0674	0,001
Discovery cohort	Type 2 diabetes	Cigarettes smoked per day	Weighted median	22	0,023	0,041	0,579	1,023	0,945	1,107					
Discovery cohort	Type 2 diabetes	Cigarettes smoked per day	Inverse variance weighted	22	0,103	0,044	0,018	1,108	1,018	1,207					
Discovery cohort	Type 2 diabetes	Cigarettes smoked per day	Simple median	22	0,156	0,059	0,008	1,169	1,042	1,311					
Discovery cohort	Type 2 diabetes	Ever smoked	MR Egger	6	1,361	2,89	0,662	3,902	0,014	1124,59	-0,0172	0,0418	0,7023	0,0009	0,0015
Discovery cohort	Type 2 diabetes	Ever smoked	Weighted median	6	0,53	0,329	0,107	1,699	0,892	3,237					
Discovery cohort	Type 2 diabetes	Ever smoked	Inverse variance weighted	6	0,189	0,416	0,649	1,208	0,534	2,731					
Discovery cohort	Type 2 diabetes	Ever smoked	Simple median	6	0,63	0,299	0,036	1,877	1,044	3,376					
Discovery cohort	Type 2 diabetes	smoking initiation	MR Egger	85	0,288	0,283	0,313	1,333	0,766	2,322	-0,0028	0,0074	0,7052	0	0
Discovery cohort	Type 2 diabetes	smoking initiation	Weighted median	85	0,141	0,05	0,004	1,152	1,045	1,27					
Discovery cohort	Type 2 diabetes	smoking initiation	Inverse variance weighted	85	0,182	0,056	0,001	1,2	1,076	1,338					
Discovery cohort	Type 2 diabetes	smoking initiation	Simple median	85	0,157	0,05	0,002	1,17	1,061	1,29					
Finngen Cohort	Type 2 diabetes	Age Of Smoking Initiation	MR Egger	7	-0,939	1,282	0,497	0,391	0,032	4,822	0,0113	0,0271	0,6952	0,0655	0,0973
Finngen Cohort	Type 2 diabetes	Age Of Smoking Initiation	Weighted median	7	-0,472	0,394	0,23	0,624	0,288	1,349					
Finngen Cohort	Type 2 diabetes	Age Of Smoking Initiation	Inverse variance weighted	7	-0,435	0,38	0,253	0,648	0,307	1,364					
Finngen Cohort	Type 2 diabetes	Age Of Smoking Initiation	Simple median	7	-0,301	0,452	0,506	0,74	0,305	1,796					
Finngen Cohort	Type 2 diabetes	Cigarettes smoked per day	MR Egger	22	0,05	0,144	0,733	1,051	0,793	1,393	0,0021	0,0096	0,8309	0	0,0001
Finngen Cohort	Type 2 diabetes	Cigarettes smoked per day	Weighted median	22	0,035	0,067	0,602	1,035	0,908	1,18					
Finngen Cohort	Type 2 diabetes	Cigarettes smoked per day	Inverse variance weighted	22	0,075	0,08	0,348	1,078	0,921	1,262					
Finngen Cohort	Type 2 diabetes	Cigarettes smoked per day	Simple median	22	0,208	0,096	0,03	1,232	1,02	1,487					
Finngen Cohort	Type 2 diabetes	Ever smoked	MR Egger	6	3,131	2,527	0,283	22,9	0,162	3244,51	-0,0431	0,0371	0,31	0,5519	0,4955
Finngen Cohort	Type 2 diabetes	Ever smoked	Weighted median	6	0,398	0,522	0,447	1,488	0,535	4,143					
Finngen Cohort	Type 2 diabetes	Ever smoked	Inverse variance weighted	6	0,233	0,403	0,563	1,262	0,573	2,78					
Finngen Cohort	Type 2 diabetes	Ever smoked	Simple median	6	0,397	0,516	0,442	1,487	0,54	4,091					
Finngen Cohort	Type 2 diabetes	smoking initiation	MR Egger	85	0,54	0,339	0,115	1,715	0,883	3,333	-0,0088	0,0088	0,3225	0,12	0,1188
Finngen Cohort	Type 2 diabetes	smoking initiation	Weighted median	85	0,18	0,089	0,044	1,197	1,005	1,427					
Finngen Cohort	Type 2 diabetes	smoking initiation	Inverse variance weighted	85	0,209	0,067	0,002	1,232	1,081	1,405					
Finngen Cohort	Type 2 diabetes	smoking initiation	Simple median	85	0,187	0,095	0,05	1,206	1	1,454					

Table S4: Genetic instruments of smoking-related DNA methylation at CpG sites used in this Mendelian randomization study

exposure	SNP	effect_allele	other_allele	beta	se	pval	F
cg01300096	rs57912571	G	A	0,2593	0,0282901	0	84
cg01300096	rs210155	C	T	0,0518	0,0097745	0	28,1
cg07123182	rs231359	A	C	-0,347	0,0097608	4,18E-276	1260,6
cg01744331	rs231359	A	C	-0,353	0,0091619	0	1486,5
cg16556677	rs231359	A	C	-0,342	0,0091835	3,72E-304	1389,7
cg10965178	rs4672249	T	C	0,0984	0,0095859	0	105,3
cg10965178	rs2708146	G	A	0,1016	0,0085766	0	140,2
cg24142464	rs372271083	A	G	-0,156	0,0135326	0	133,5
cg26963277	rs231360	T	C	-0,258	0,0094586	0	742,9
cg26963277	rs6578283	G	A	0,11	0,0109168	0	101,5
cg09287933	rs116647495	C	G	0,4579	0,0330467	1,16E-43	192
cg23657179	rs1897191	A	G	0,33	0,0092376	1,51E-279	1276,4
cg23657179	rs56134657	A	G	0,3117	0,0193455	2,15E-58	259,6
cg23657179	rs5752598	A	C	-0,245	0,0118735	6,17E-95	427,3
cg12864721	rs56134657	A	G	0,3039	0,0193468	1,30E-55	246,8
cg12864721	rs1897191	A	G	0,2682	0,0093245	7,01E-182	827,1
cg12864721	rs5752598	A	C	-0,223	0,0120013	4,38E-77	345,3
cg10672416	rs4275659	T	C	-0,366	0,0093201	0	1542,4
cg15948030	rs3129986	T	C	-0,103	0,0160651	0	41,5
cg15948030	rs79611767	C	T	0,0947	0,0141315	0	44,9
cg23756272	rs3810027	G	C	0,26	0,0091192	8,86E-179	812,8
cg20698113	rs137856	A	G	-0,139	0,0088625	1,58E-55	246,4
cg11801110	rs137856	A	G	-0,181	0,008833	6,01E-93	418,2
cg09861057	rs61812654	A	G	0,1404	0,0121862	0	132,8
cg15182635	rs4746268	G	T	0,3002	0,026268	0	130,6
cg15182635	rs5752598	A	C	-0,246	0,0119428	3,16E-94	424,1
cg15182635	rs1897191	A	G	0,2854	0,0092977	6,29E-207	942,3
cg03599224	rs1121800	A	T	-0,263	0,0091167	1,76E-183	834,4
cg03599224	rs2507993	C	T	-0,086	0,0143481	1,70E-09	36,3
cg03864215	rs7928810	C	A	0,3884	0,0087822	0	1956,2
cg03864215	rs4148600	C	T	-0,099	0,0108183	8,10E-20	83
cg20069688	rs3130909	C	T	-0,52	0,0134233	0	1501,7
cg17478749	rs3094124	C	G	-0,102	0,0201991	0	25,5
cg17478749	rs61740337	G	A	0,2013	0,0193676	0	108
cg17478749	rs10947131	T	C	0,1114	0,0142752	0	60,9

Table S5: Two-sample Mendelian randomization of smoking-related DNA methylation (CpG sites) and the risk of type 2 diabetes

outcome	exposure	location	gene	method	nsnp	beta	se	pval	lo_ci	up_ci	or	or_lci95	or_uci95	pvalR
Type 2 diab	cg01300096	chr6:33384490	CUTA	Inverse variance weighted	2	0,489395	0,051936	4,38E-21	0,3876	0,59119	1,631329	1,47344	1,806137	4,38E-21
Type 2 diab	cg07123182	chr11:2722391	KCNQ1OT1; KCNQ1	Wald ratio	1	-0,18323	0,021642	2,53E-17	-0,22565	-0,14082	0,832574	0,797997	0,86865	2,53E-17
Type 2 diab	cg01744331	chr11:2722358	KCNQ1OT1; KCNQ1	Wald ratio	1	-0,17976	0,021232	2,53E-17	-0,22138	-0,13815	0,835468	0,801414	0,870969	2,53E-17
Type 2 diab	cg16556677	chr11:2722401	KCNQ1OT1; KCNQ1	Wald ratio	1	-0,18549	0,021908	2,53E-17	-0,22843	-0,14255	0,830701	0,795786	0,867147	2,53E-17
Type 2 diab	cg10965178	chr1:43766752	TIE1	Inverse variance weighted	2	0,33657	0,048811	5,37E-12	0,240901	0,432239	1,400137	1,272395	1,540704	5,37E-12
Type 2 diab	cg24142464	chr1:40040489	PABPC4	Wald ratio	1	-0,34344	0,052443	5,80E-11	-0,44623	-0,24065	0,709328	0,640039	0,786117	5,80E-11
Type 2 diab	cg26963277	chr11:2722407	KCNQ1OT1; KCNQ1	Inverse variance weighted	2	-0,1836	0,030115	1,08E-09	-0,24263	-0,12458	0,832268	0,784564	0,882872	1,08E-09
Type 2 diab	cg09287933	chr6:33384473	CUTA	Wald ratio	1	0,301362	0,054376	2,99E-08	0,194785	0,407939	1,351698	1,215049	1,503716	2,99E-08
Type 2 diab	cg23657179	chr10:77165025	C10orf41	Inverse variance weighted	3	0,088384	0,015999	3,31E-08	0,057025	0,119743	1,092407	1,058682	1,127207	3,31E-08
Type 2 diab	cg12864721	chr10:77164987	C10orf41	Inverse variance weighted	3	0,103245	0,018833	4,20E-08	0,066333	0,140157	1,108763	1,068582	1,150455	4,20E-08
Type 2 diab	cg10672416	chr12:123718706	C12orf65	Wald ratio	1	0,099991	0,018851	1,13E-07	0,063043	0,136938	1,105161	1,065073	1,146758	1,13E-07
Type 2 diab	cg15948030	chr6:31760825	VARS	Inverse variance weighted	2	-0,43768	0,083449	1,56E-07	-0,60124	-0,27412	0,645534	0,548134	0,760242	1,56E-07
Type 2 diab	cg23756272	chr18:60904418	BCL2	Wald ratio	1	0,127699	0,025001	3,26E-07	0,078697	0,176702	1,136211	1,081876	1,193276	3,26E-07
Type 2 diab	cg20698113	chr22:50357214	PIM3	Wald ratio	1	0,23218	0,046724	6,72E-07	0,140602	0,323758	1,261347	1,150966	1,382313	6,72E-07
Type 2 diab	cg11801110	chr22:50356763	PIM3	Wald ratio	1	0,178815	0,035984	6,72E-07	0,108285	0,249344	1,195799	1,114366	1,283184	6,72E-07
Type 2 diab	cg09861057	chr1:154492947	TDRD10	Wald ratio	1	0,318323	0,066941	1,98E-06	0,18712	0,449527	1,374821	1,205772	1,56757	1,98E-06
Type 2 diab	cg15182635	chr10:77165167	C10orf41	Inverse variance weighted	3	0,09217	0,019467	2,19E-06	0,054015	0,130325	1,096551	1,055501	1,139199	2,19E-06
Type 2 diab	cg03599224	chr6:31541349	LTA	Inverse variance weighted	2	0,110126	0,023446	2,64E-06	0,064172	0,156079	1,116418	1,066276	1,168919	2,64E-06
Type 2 diab	cg03864215	chr11:17408437	KCNJ11	Inverse variance weighted	2	0,124639	0,027857	7,67E-06	0,070039	0,179239	1,13274	1,07255	1,196306	7,67E-06
Type 2 diab	cg20069688	chr6:31941049	STK19; DOM3Z	Wald ratio	1	-0,11131	0,025376	1,15E-05	-0,16105	-0,06157	0,894662	0,851253	0,940286	1,15E-05
Type 2 diab	cg17478749	chr6:31589365	SNORA38; BAT2	Inverse variance weighted	3	0,224396	0,051186	1,17E-05	0,124072	0,32472	1,251566	1,132097	1,383643	1,17E-05

Table S6: Heterogeneity test results (Cochran's Q statistics) for MR analyses

Cohort	outcome	exposure	method	Q	Q_df	Q_pval
Discovery cohort	Type 2 diabetes	Cigarettes smoked per day	MR Egger	30,15262	20	0,067418
Discovery cohort	Type 2 diabetes	Cigarettes smoked per day	Inverse variance weighted	46,94031	21	0,000957
Discovery cohort	Type 2 diabetes	Age Of Smoking Initiation	MR Egger	0,8756	4	0,928035
Discovery cohort	Type 2 diabetes	Age Of Smoking Initiation	Inverse variance weighted	1,66603	5	0,893151
Discovery cohort	Type 2 diabetes	Ever smoked	MR Egger	18,77975	4	0,000868
Discovery cohort	Type 2 diabetes	Ever smoked	Inverse variance weighted	19,57217	5	0,001503
Discovery cohort	Type 2 diabetes	smoking initiation	MR Egger	265,6872	83	5,51E-21
Discovery cohort	Type 2 diabetes	smoking initiation	Inverse variance weighted	266,1485	84	8,47E-21
Finngen Cohort	Type 2 diabetes	Cigarettes smoked per day	MR Egger	54,61882	20	4,68E-05
Finngen Cohort	Type 2 diabetes	Cigarettes smoked per day	Inverse variance weighted	54,7467	21	7,70E-05
Finngen Cohort	Type 2 diabetes	Age Of Smoking Initiation	MR Egger	10,36502	5	0,065529
Finngen Cohort	Type 2 diabetes	Age Of Smoking Initiation	Inverse variance weighted	10,72238	6	0,097345
Finngen Cohort	Type 2 diabetes	Ever smoked	MR Egger	3,035281	4	0,551939
Finngen Cohort	Type 2 diabetes	Ever smoked	Inverse variance weighted	4,384489	5	0,495486
Finngen Cohort	Type 2 diabetes	smoking initiation	MR Egger	98,33706	83	0,119978
Finngen Cohort	Type 2 diabetes	smoking initiation	Inverse variance weighted	99,51086	84	0,118844

STROBE-MR checklist of recommended items to address in reports of Mendelian randomization studies[1 2]

Item No.	Section	Checklist item	Page No.	Relevant text from manuscript
1	**TITLE and ABSTRACT**	Indicate Mendelian randomization (MR) as the study's design in the title and/or the abstract if that is a main purpose of the study	2	In this study, we employed a two-sample Mendelian randomization (MR) analysis to investigate the effects of various smoking behaviors (including smoking initiation, age of smoking initiation, cigarettes per day, and ever smoked) and methylation at smoking-related CpG sites on the risk of developing T2D.
	**INTRODUCTION**			
2	**Background**	Explain the scientific background and rationale for the reported study. What is the exposure? Is a potential causal relationship between exposure and outcome plausible? Justify why MR is a helpful method to address the study question	3	Numerous observational studies have demonstrated that smokers exhibit a heightened risk of developing T2D compared to non-smokers<br>In this study, we employed a two-sample MR analysis to investigate the relationship between genetic predisposition to smoking behaviors and the risk of T2D, as well as to assess the influence of genetically predicted methylations at smoking-related CpG sites on T2D risk. Additionally, we corroborated our findings through genetic co-localization analysis.<br><br>Exposure: smoking initiation<br><br>Nevertheless, due to the association of smoking with various lifestyle and socioeconomic factors, and the inherent challenges in controlling for all confounding variables in observational studies, the precise causal relationship between smoking and T2D remains to be inadequately understood.<br>Mendelian randomization (MR) is an epidemiological method based on genetic variants. MR analysis uses genetic variants to determine the causal effect of the risk factor on the outcome, which was considered to be advantageous to observational studies because it avoids confounding factors and reverse causation.
3	**Objectives**	State specific objectives clearly, including pre-specified causal hypotheses (if any). State that MR is a method that, under specific assumptions, intends to estimate causal effects	3-4	Mendelian randomization (MR) is an epidemiological method based on genetic variants. MR analysis uses genetic variants to determine the causal effect of the risk factor on the outcome, which

				was considered to be advantageous to observational studies because it avoids confounding factors and reverse causation.<br>In this study, we employed a two-sample MR analysis to investigate the relationship between genetic predisposition to smoking behaviors and the risk of T2D, as well as to assess the influence of genetically predicted methylations at smoking-related CpG sites on T2D risk.
	**METHODS**			
4	**Study design and data sources**	Present key elements of the study design early in the article. Consider including a table listing sources of data for all phases of the study. For each data source contributing to the analysis, describe the following:		
	a)	Setting: Describe the study design and the underlying population, if possible. Describe the setting, locations, and relevant dates, including periods of recruitment, exposure, follow-up, and data collection, when available.	28	Figure1: study design<br>Table S1: Datasets used in the study
	b)	Participants: Give the eligibility criteria, and the sources and methods of selection of participants. Report the sample size, and whether any power or sample size calculations were carried out prior to the main analysis	28	Figure1: study design<br>Table S1: Datasets used in the study
	c)	Describe measurement, quality control and selection of genetic variants	28	Figure1: study design
	d)	For each exposure, outcome, and other relevant variables, describe methods of assessment and diagnostic criteria for diseases		
	e)	Provide details of ethics committee approval and participant informed consent, if relevant		Our study was a secondary analysis of publicly available data. Informed consent was obtained from all participants as per the original GWAS protocols, and all ethical approvals for the GWAS were obtained by the original GWAS authors.
5	**Assumptions**	Explicitly state the three core IV assumptions for the main analysis (relevance, independence and exclusion restriction) as well assumptions for any additional or sensitivity analysis	4	The MR analysis was conducted under three principal assumptions: (1) Relevance: The instrumental variables (IVs) must exhibit an association with the exposure, specifically smoking behaviors. (2) Exclusivity: The IVs should exert an influence on the outcome (type 2 diabetes), solely through their impact on the exposure. (3) Independence: The IVs must remain independent of any confounding variables that affect both the exposure and the outcome.

6	**Statistical methods: main analysis**	Describe statistical methods and statistics used	8	The primary analytical approach employed was the inverse variance weighted (IVW) method under a multiplicative random effects model. This random-effects IVW method presupposes that all genetic variants serve as valid instrumental variables and addresses potential heterogeneity through the application of Wald ratio estimates. The results were presented as odds ratios (ORs) accompanied by 95% confidence intervals (CIs), indicating the risk of the disease per one standard deviation (SD) alteration in genetically predicted smoking behaviors or smoking-related DNA methylation at CpG sites. To address the issue of multiple testing, the Bonferroni correction was employed, and associations were deemed statistically significant if they had an adjusted p-value of less than 0.05, as determined by either the random-effects inverse-variance weighted (IVW) model or the Wald ratio model.<br><br>To evaluate the third assumption of MR (the exclusion restriction criterion), we employed MR-Egger regression to identify potential violations arising from directional horizontal pleiotropy. Furthermore, we utilized the weighted median method, which yields consistent estimates even when up to 50% of the instrumental variables are affected by pleiotropy. Heterogeneity was examined using Cochran's Q test in both the IVW and MR-Egger methods.<br><br>For results that passed our MR significance threshold (adjusted p-value < 0.05), we conducted a co-localization analysis utilizing the COLOC package in R. Co-localization analysis is instrumental in assessing whether a shared causal variant (PP.H4) is present between the disease genome-wide association study (GWAS) and the instruments for smoking-related DNA methylation at CpG sites. The analysis was executed using default priors (prior probability of association = 1×10^{-4},

				prior probability of shared causal variant = 1×10^{-5}). A PP.H4 exceeding 85% was considered indicative of strong evidence for co-localization.
		a) Describe how quantitative variables were handled in the analyses (i.e., scale, units, model)		
		b) Describe how genetic variants were handled in the analyses and, if applicable, how their weights were selected	6-7	In MR analyses, the genetic variants employed as instrumental variables for the exposure must be uncorrelated and exhibit a strong association ($p < 5\times10^{-8}$) with the exposure of interest. From the SNPs identified in the GWAS of smoking behaviors and mQTLs, we further refined our selection to include only those variants with a minor allele frequency (MAF) > 0.01. Additionally, we ensured that these SNPs were not in linkage disequilibrium (LD) ($r^2 < 0.001$ and clumping window within 10,000 base pairs, based on the European 1000 Genomes Project reference panel). This was done to meet the assumption of independence among the instrumental variables used in MR analyses. To mitigate the risk of weak instrument bias, we computed the F statistic for each instrumental variable, adhering to the widely accepted threshold of $F > 10$. If a selected SNP from the first step was absent in the outcome data, we used its proxy SNP with $r^2 > 0.8$ instead. When multiple proxy SNPs were available, the proxy with the highest r^2 and the lowest p-value in relation to the exposure was selected.
		c) Describe the MR estimator (e.g. two-stage least squares, Wald ratio) and related statistics. Detail the included covariates and, in case of two-sample MR, whether the same covariate set was used for adjustment in the two samples	8	
		d) Explain how missing data were addressed		
		e) If applicable, indicate how multiple testing was addressed	8	
7	**Assessment of assumptions**	Describe any methods or prior knowledge used to assess the assumptions or justify their validity	6-7	In MR analyses, the genetic variants employed as instrumental variables for the exposure must be uncorrelated and exhibit a strong association ($p < 5\times10^{-8}$) with the exposure of interest. From the SNPs identified in the GWAS of smoking behaviors and mQTLs, we further refined our selection to include only those variants with a minor allele

			frequency (MAF) > 0.01. Additionally, we ensured that these SNPs were not in linkage disequilibrium (LD) ($r^2 < 0.001$ and clumping window within 10,000 base pairs, based on the European 1000 Genomes Project reference panel). This was done to meet the assumption of independence among the instrumental variables used in MR analyses. To mitigate the risk of weak instrument bias, we computed the F statistic for each instrumental variable, adhering to the widely accepted threshold of $F > 10$. If a selected SNP from the first step was absent in the outcome data, we used its proxy SNP with $r^2 > 0.8$ instead. When multiple proxy SNPs were available, the proxy with the highest r^2 and the lowest p-value in relation to the exposure was selected.
8	**Sensitivity analyses and additional analyses**	Describe any sensitivity analyses or additional analyses performed (e.g. comparison of effect estimates from different approaches, independent replication, bias analytic techniques, validation of instruments, simulations)	The primary analytical approach employed was the inverse variance weighted (IVW) method under a multiplicative random effects model. This random-effects IVW method presupposes that all genetic variants serve as valid instrumental variables and addresses potential heterogeneity through the application of Wald ratio estimates. The results were presented as odds ratios (ORs) accompanied by 95% confidence intervals (CIs), indicating the risk of the disease per one standard deviation (SD) alteration in genetically predicted smoking behaviors or smoking-related DNA methylation at CpG sites. To address the issue of multiple testing, the Bonferroni correction was employed, and associations were deemed statistically significant if they had an adjusted p-value of less than 0.05, as determined by either the random-effects inverse-variance weighted (IVW) model or the Wald ratio model.<br><br>To evaluate the third assumption of MR (the exclusion restriction criterion), we employed MR-Egger regression to identify potential violations arising from directional horizontal pleiotropy. Furthermore, we utilized the weighted median method, which yields consistent estimates even when up to 50% of the instrumental variables are

			affected by pleiotropy. Heterogeneity was examined using Cochran's Q test in both the IVW and MR-Egger methods.
9	**Software and pre-registration**		
	a) Name statistical software and package(s), including version and settings used	8	We used R Software 3.6.1 software (https://www.r-project.org/) and the "COLOC package" package (https://mrcieu.github.io/TwoSampleMR/) to conduct MR analysis.
	b) State whether the study protocol and details were pre-registered (as well as when and where)		The study protocol and details have not been pre-registered.
	**RESULTS**		
10	**Descriptive data**		
	a) Report the numbers of individuals at each stage of included studies and reasons for exclusion. Consider use of a flow diagram	5-6	We obtained summary statistics for type 2 diabetes from 32 genome-wide association studies (GWAS) conducted among individuals of European ancestry (Table S1), which encompassed 74,124 cases and 824,006 controls[25]<br>Additionally, we included two additional datasets for replication. The first dataset was sourced from the FinnGen consortium, comprising 17,268 cases of type 2 diabetes and 184,778 controls. The second dataset utilized in this study was obtained from BioBank Japan (BBJ). We included data comprising 40,250 cases of type 2 diabetes and 170,615 control subjects from the BBJ dataset[28].
	b) Report summary statistics for phenotypic exposure(s), outcome(s), and other relevant variables (e.g. means, SDs, proportions)		Table S1-5
	c) If the data sources include meta-analyses of previous studies, provide the assessments of heterogeneity across these studies		The data sources do not include meta-analyses.
	d) For two-sample MR:<br>i. Provide justification of the similarity of the genetic variant-exposure associations between the exposure and outcome samples<br>ii. Provide information on the number of individuals who overlap between the exposure and outcome studies		Table S2-4
11	**Main results**		

		a)	Report the associations between genetic variant and exposure, and between genetic variant and outcome, preferably on an interpretable scale		Table S2-4
		b)	Report MR estimates of the relationship between exposure and outcome, and the measures of uncertainty from the MR analysis, on an interpretable scale, such as odds ratio or relative risk per SD difference	28-29	Table 1; Table S5; Figure 2; Figure 3
		c)	If relevant, consider translating estimates of relative risk into absolute risk for a meaningful time period		
		d)	Consider plots to visualize results (e.g. forest plot, scatterplot of associations between genetic variants and outcome versus between genetic variants and exposure)	28-29	Figure 2; Figure 3
12	**Assessment of assumptions**				
		a)	Report the assessment of the validity of the assumptions	6	In MR analyses, the genetic variants employed as instrumental variables for the exposure must be uncorrelated and exhibit a strong association ($p < 5\times10^{-8}$) with the exposure of interest. From the SNPs identified in the GWAS of smoking behaviors and mQTLs, we further refined our selection to include only those variants with a minor allele frequency (MAF) > 0.01. Additionally, we ensured that these SNPs were not in linkage disequilibrium (LD) ($r^2 < 0.001$ and clumping window within 10,000 base pairs, based on the European 1000 Genomes Project reference panel). This was done to meet the assumption of independence among the instrumental variables used in MR analyses. To mitigate the risk of weak instrument bias, we computed the F statistic for each instrumental variable, adhering to the widely accepted threshold of $F > 10$. If a selected SNP from the first step was absent in the outcome data, we used its proxy SNP with $r^2 > 0.8$ instead. When multiple proxy SNPs were available, the proxy with the highest r^2 and the lowest p-value in relation to the exposure was selected.
		b)	Report any additional statistics (e.g., assessments of heterogeneity across genetic variants, such as I^2, Q statistic or E-value)		Table S3
13	**Sensitivity analyses and additional analyses**				

		a) Report any sensitivity analyses to assess the robustness of the main results to violations of the assumptions		Table S3
		b) Report results from other sensitivity analyses or additional analyses		Table S2-4
		c) Report any assessment of direction of causal relationship (e.g., bidirectional MR)		
		d) When relevant, report and compare with estimates from non-MR analyses		
		e) Consider additional plots to visualize results (e.g., leave-one-out analyses)		Table S2-4
	**DISCUSSION**			
14	**Key results**	Summarize key results with reference to study objectives	13	Our genomic analysis provided robust evidence supporting the pathogenic impact of smoking initiation on T2D.<br><br>When exploring the effect of genetically predicted methylations at smoking-related CpG sites on T2D risk, we found that methylations at 21 CpG sites regulated T2D risk through epigenetic modification, of which methylations at 14 CpG sites were associated with an increased risk of T2D, while methylations at the remaining seven CpG sites were associated with a decreased risk of T2D. Among these CpG sites, 14 of which were replicated in the FinnGen database, and 12 of which were replicated in the BBJ database. In addition, we performed genetic co-localization analysis and found that four CpG sites, including cg23756272 (*BCL2*), cg0386421 (*KCNJ11*), cg09861057 (*TDRD10*), and cg10672416 (*C12orf65*), had strong co-localization evidence.
15	**Limitations**	Discuss limitations of the study, taking into account the validity of the IV assumptions, other sources of potential bias, and imprecision. Discuss both direction and magnitude of any potential bias and any efforts to address them	18-19	There are also some limitations to our study. Firstly, the DNA methylation data for smoking-related CpG sites were derived from cross-sectional EWAS, which precludes the assessment of the temporal progression, kinetics, and dynamics of smoking's impact on DNA methylation. Consequently, longitudinal studies are warranted to elucidate the causal relationship between smoking and DNA methylation over time. Secondly, the analysis was based on smoking-related DNA methylation patterns in blood samples. It is important to

					acknowledge that DNA methylation characteristics can vary significantly across different tissues. Furthermore, in contrast to CpG sites, CpG islands are genomic regions characterized by a higher density of CpG dinucleotides. These islands are typically situated near gene promoters and are pivotal in gene regulation, particularly concerning DNA methylation. Consequently, the analysis of CpG islands, as opposed to CpG sites, may offer more comprehensive insights into the epigenetic regulation of genes. This is because alterations in methylation patterns across CpG islands can substantially influence gene expression. In our study, we could acquire DNA methylation data for smoking-related CpG sites, but not for smoking-related CpG islands. Therefore, the analysis of CpG islands could not be replicated in this investigation. Future research could incorporate these MR analyses once the necessary data become accessible.
16	**Interpretation**				
		a)	Meaning: Give a cautious overall interpretation of results in the context of their limitations and in comparison with other studies	19	In conclusion, this study offered significant insights into the potential pathogenic role of smoking in the development of T2D. Our findings corroborated a definitive association between smoking and T2D risk. Furthermore, the evidence indicated that alterations in DNA methylation at specific CpG sites and associated genes played a crucial role in mediating this relationship.
		b)	Mechanism: Discuss underlying biological mechanisms that could drive a potential causal relationship between the investigated exposure and the outcome, and whether the gene-environment equivalence assumption is reasonable. Use causal language carefully, clarifying that IV estimates may provide causal effects only under certain assumptions	12	Several potential mechanisms may elucidate the causal relationship between smoking and the risk of T2D. Nicotine, the primary bioactive constituent of cigarettes, could elevate blood glucose levels by activating the sympathetic components of the autonomic nervous system. It has been shown that nicotine increases the production rate of glycated hemoglobin by 34%. Additionally, nicotine could impair the function and quality of pancreatic β-cells, thereby disrupting their feedback regulation and impairing glucose homeostasis, which is a critical factor in the pathogenesis of T2D. Moreover, chronic inflammation serves as a significant

				predisposing factor for T2D, and the inflammation induced by smoking partially elucidated the causal relationship between smoking and T2D.
		c) Clinical relevance: Discuss whether the results have clinical or public policy relevance, and to what extent they inform effect sizes of possible interventions	13	Therefore, considering the detrimental impact of smoking on T2D, smoking cessation is of paramount importance.
17	**Generalizability**	Discuss the generalizability of the study results (a) to other populations, (b) across other exposure periods/timings, and (c) across other levels of exposure	18	Additionally, we investigated the association between genetically predicted methylation at smoking-related CpG sites and T2D risk across diverse independent datasets, thereby augmenting the generalizability of the population and the validity of our findings.
	**OTHER INFORMATION**			
18	**Funding**	Describe sources of funding and the role of funders in the present study and, if applicable, sources of funding for the databases and original study or studies on which the present study is based	19	Not applicable.
19	**Data and data sharing**	Provide the data used to perform all analyses or report where and how the data can be accessed, and reference these sources in the article. Provide the statistical code needed to reproduce the results in the article, or report whether the code is publicly accessible and if so, where	19	Datasets used or analyzed during this study are available upon reasonable request from the corresponding author.
20	**Conflicts of Interest**	All authors should declare all potential conflicts of interest	19	The authors have no relevant financial or non-financial interests to disclose.

This checklist is copyrighted by the Equator Network under the Creative Commons Attribution 3.0 Unported (CC BY 3.0) license.

1. Skrivankova VW, Richmond RC, Woolf BAR, Yarmolinsky J, Davies NM, Swanson SA, et al. Strengthening the Reporting of Observational Studies in Epidemiology using Mendelian Randomization (STROBE-MR) Statement. JAMA. 2021;under review.

2. Skrivankova VW, Richmond RC, Woolf BAR, Davies NM, Swanson SA, VanderWeele TJ, et al. Strengthening the Reporting of Observational Studies in Epidemiology using Mendelian Randomisation (STROBE-MR): Explanation and Elaboration. BMJ. 2021;375:n2233.
